# Genomic epidemiology and transmission dynamics of recurrent *Clostridioides difficile* infection in Western Australia

**DOI:** 10.1007/s10096-023-04569-x

**Published:** 2023-03-20

**Authors:** Daniel R. Knight, Korakrit Imwattana, Deirdre A. Collins, Su-Chen Lim, Stacey Hong, Papanin Putsathit, Thomas V. Riley

**Affiliations:** 1 School of Biomedical Sciences, The University of Western Australia, Nedlands, Western Australia Australia; 2 Department of Microbiology, PathWest Laboratory Medicine, Queen Elizabeth II Medical Centre, Nedlands, Western Australia Australia; 3 Department of Microbiology, Faculty of Medicine Siriraj Hospital, Mahidol University, Bangkok, Thailand; 4 School of Medical and Health Sciences, Edith Cowan University, Joondalup, Western Australia Australia; 5 Communicable Disease Control Directorate, Western Australia Department of Health, East Perth, Perth, Western Australia Australia; 6 Harry Butler Institute, Murdoch University, Murdoch, Western Australia Australia

**Keywords:** *Clostridioides difficile*, Molecular epidemiology, Recurrence, Relapse, Reinfection, Transmission

## Abstract

Recurrent cases of *Clostridioides difficile* infection (rCDI) remain one of the most common and serious challenges faced in the management of CDI. The accurate distinction between a relapse (caused by infection with the same strain) and reinfection (caused by a new strain) has implications for infection control and prevention, and patient therapy. Here, we used whole-genome sequencing to investigate the epidemiology of 94 *C. difficile* isolates from 38 patients with rCDI in Western Australia. The *C. difficile* strain population comprised 13 sequence types (STs) led by ST2 (PCR ribotype (RT) 014, 36.2%), ST8 (RT002, 19.1%) and ST34 (RT056, 11.7%). Among 38 patients, core genome SNP (cgSNP) typing found 27 strains (71%) from initial and recurring cases differed by ≤ 2 cgSNPs, suggesting a likely relapse of infection with the initial strain, while eight strains differed by ≥ 3 cgSNPs, suggesting reinfection. Almost half of patients with CDI relapse confirmed by WGS suffered episodes that occurred outside the widely used 8-week cut-off for defining rCDI. Several putative strain transmission events between epidemiologically unrelated patients were identified. Isolates of STs 2 and 34 from rCDI cases and environmental sources shared a recent evolutionary history, suggesting a possible common community reservoir. For some rCDI episodes caused by STs 2 and 231, within-host strain diversity was observed, characterised by loss/gain of moxifloxacin resistance. Genomics improves discrimination of relapse from reinfection and identifies putative strain transmission events among patients with rCDI. Current definitions of relapse and reinfection based on the timing of recurrence need to be reconsidered.

## Introduction

*Clostridioides difficile* infection (CDI) has become more common, severe and difficult to treat in recent years [1]. Recurrent CDI (rCDI), where symptoms of CDI return following initial resolution, remains a common and serious challenge in the management of CDI. rCDI is a significant factor contributing to CDI-associated morbidity, causing substantial stress to patients and impacting healthcare systems [2–4]. Up to 30% of patients with an initial episode of CDI experience at least one symptomatic recurrence following the discontinuation of therapy, and up to 45% and 65% of those go on to develop second and third recurrences, respectively [5, 6].

The development of rCDI is influenced by a combination of host and pathogen factors. Many factors are the same antecedents that resulted in the initial CDI episode—dysbiosis of the colonic microbiota, inadequate host immune response to *C. difficile* toxins, co-morbidities and prolonged hospital stays [2]. Other risk factors for rCDI include advanced age, concurrent antimicrobial usage with CDI therapy, leukocytosis, hypoalbuminemia, elevated creatinine, renal failure and use of proton-pump inhibitors [4]. Due to a higher rate of therapeutic failure, initial infection with hypervirulent *C. difficile* strains has been linked to more frequent recurrences [7]. Accordingly, prolonged and complicated treatment regimens for rCDI result in extended hospitalization with associated costs. The annual cost of rCDI in the USA was estimated to be US $2.8 billion [3].

rCDI can be subdivided into relapse (CDI caused by a new infection with the same endogenous initial strain) or reinfection (CDI caused by one or more different strains acquired from an exogenous source) [8]. Clinical practice guidelines recommend that if the time elapsed between two episodes of CDI is > 8 weeks and that prior symptoms have resolved with or without therapy, then, the second episode is classed as a new infection as opposed to a recurrent infection [9]. Recent phase III clinical trials for bezlotoxumab (MODIFY I and II) used a longer, 12-week cut-off to define rCDI [10, 11].

This distinction between relapse and reinfection is critical. Over- or underestimation of rCDI has implications for surveillance, patient treatment, infection prevention and control and clinical trials investigating the effectiveness of novel therapies [3, 4, 12]. Conventional typing approaches such as PCR ribotyping do not provide sufficient resolution to detect subtle within-strain diversity and contribute to inaccurate epidemiological characterisation of rCDI [13, 14]. In this study, whole-genome sequencing (WGS) was used to investigate the genomic epidemiology, antimicrobial resistance (AMR) and environmental origins of rCDI in three hospitals in Perth, Western Australia (WA).

## Materials and methods

### Study population

**Fig. 1:**
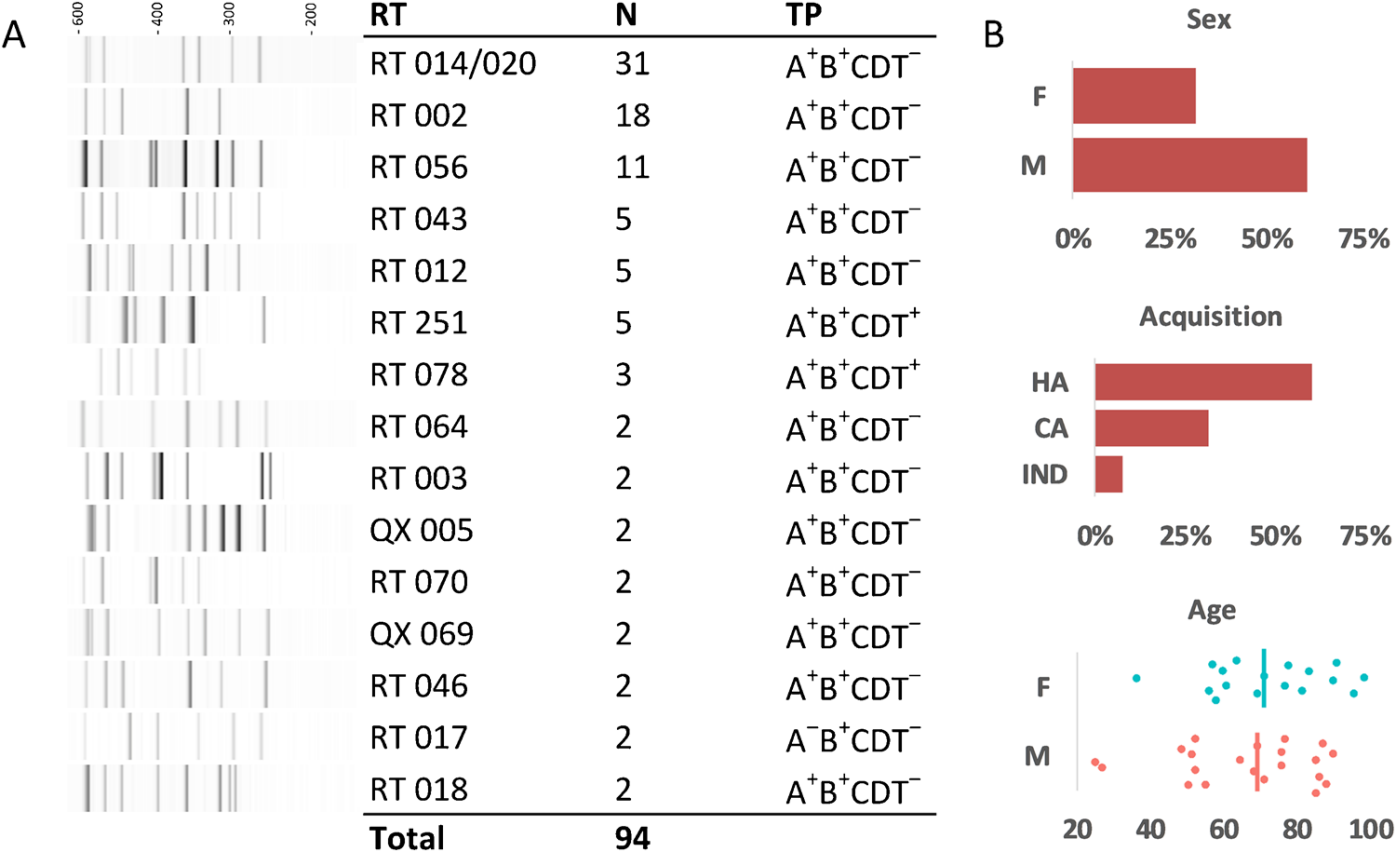
Isolate molecular epidemiology and patient demographics. **A** PCR ribotyping patterns (16S-23S rRNA ISR sequences) for 15 unique RTs analysed in this study (*n* = 94 isolates; RT, PCR ribotype; QX, novel RT assignment; TP, toxin gene profile). **B** Patient cohort metadata for sex, CDI acquisition, and age distribution (F, female; M, male; HA, hospital-associated; CA, community-associated; IND, indeterminate; vertical bar indicates median age)

The Healthcare Infection Surveillance Western Australia (HISWA) program monitors and reports on hospital-identified CDI across all acute care private and public hospitals in WA. Analysis of the HISWA dataset between July 2012 and June 2014 [15] identified 58 patients with rCDI across three hospitals in the North Metropolitan Health Service (NMHS) in Perth. CDI cases were defined as having diarrhoea with a positive faecal PCR for *tcdB* using the BD GeneOhm™ or BD MAX™ platforms. For this study, and in line with recent phase III MODIFY trials [10, 11], rCDI was defined as two or more episodes of diarrhoea accompanied by a positive *C. difficile* stool assay within a 12-week period. *C. difficile* isolates from rCDI patients were characterised by PCR ribotype (RT) and toxin genotype (*tcdA*, *tcdB* and *cdtA/B*) as previously described [16]. rCDI episodes were then determined to be either relapses or reinfections by comparing the RTs causing initial and subsequent episodes. Of these 58 patients, 38 (65%) experienced apparent relapses (CDI recurrence caused by isolates of the same RT as the initial episode). To investigate the genomic epidemiology of these apparent relapses, 94 isolates (from 38 initial episodes and 56 cumulative recurrences) belonging to 15 RTs (Fig. 1) were selected for WGS. Demographic data were collected retrospectively and relevant ethical approval for review of medical records was obtained from the Sir Charles Gairdner and Osborne Park Health Care Group Human Research Ethics Committee (#RGS0000001863).

### Whole-genome sequencing and in silico genotyping

Nextera XT libraries were prepared using genomic DNA extracted from a 48 h blood agar culture of *C. difficile* using a QuickGene DNA tissue kit (Kurabo Industries, Osaka, Japan). Libraries were sequenced using an Illumina HiSeq (Illumina, San Diego, CA, USA) to an average read depth of 67×. WGS data have been submitted to the NCBI Short Read Archive (PRJNA880992, Supplementary Data). *C. difficile* genomes were assembled and annotated with SPAdes v3.15.5 and Prokka v1.14.5, respectively. Assembled genomes (*n* = 94) are provided in Supplementary Data hosted at 10.6084/m9.figshare.20579976.v3. Genomes were screened for *C. difficile* toxin genes using Abricate v1.0 (https://github.com/tseemann/abricate). Multi-locus sequence type (MLST) and AMR genotype were determined using SRST2 v0.2.0 (https://github.com/katholt/srst2). A neighbour-joining phylogeny of MLST alleles was produced using MEGA v7 (https://www.megasoftware.net/) and iToL v6 [https://itol.embl.de/).

### Microevolutionary analysis

Core-genome single nucleotide polymorphism (cgSNP) analysis followed the gold-standard approach of Eyre et al. [17] using the haploid variant calling pipeline Snippy v4.6.0 (https://github.com/tseemann/snippy). Briefly, BWA-MEM v0.7.17 was used to map trimmed reads to finished chromosomes of phylogenetically appropriate *C. difficile* reference genomes: strains CD630 (ST54, clade 1, accession AM180355), R20291 (ST1, clade 2, accession FN545816), M68 (ST37, clade 4, accession FN668375) and M120 (ST11, clade 5, accession NC017174). Variant calling was performed for each ST-grouping using Freebayes v1.3.6 (https://github.com/freebayes) with candidate cgSNPs subsequently filtered for quality, coverage, indels, repetitive regions, mobile genetic elements and recombinative regions using vcftools, samtools and SnpEff, as previously described [18], generating a final ST-specific set of concatenated cgSNPs in clonal frame. Pairwise cgSNP differences between strains were calculated using snp-dists v0.8.2 (https://github.com/tseemann/snp-dists). Recurrent CDI cases were characterised as either a relapse with the same strain (defined as isolates differing by ≤ 2 cgSNPs) or reinfection with a new strain (defined as isolates differing by ≥ 3 cgSNPs).

### Antimicrobial susceptibility testing

Minimum inhibitory concentrations for vancomycin, metronidazole, moxifloxacin, tetracycline and clindamycin were determined using Etest methodology [19]. Clinical breakpoints followed the recommendations of CLSI and EUCAST [20, 21].

## Results

### Cohort characteristics

Demographic details of the 38 patients with histories of rCDI are presented in Table 1. Among this cohort, 55% were male, with a median age of 71 years (range: 22–100 years). Patients suffered between two and six CDI episodes (median 2) and the time to recurrence ranged from 7 days to more than 2 years (754 days) with a median of 41 days. Notably, among the 56 relapses, 53.6% and 37.5% occurred more than 60 days and 90 days, respectively, after the initial episode. Among the 38 patients, and based on the classification of initial episodes, community-associated CDI (disease onset less than 48 h after admission and more than 12 weeks after the previous hospitalisation) accounted for 31.6% of cases, hospital-associated CDI (disease onset more than 48 h after admission) accounted for 60.5% and 7.9% of cases were indeterminate (disease did not fit either CA- or HA-CDI definition).

**Table 1:** Patient demographics and clinical parameters

Clinical parameter	Result
Total number of patients	38
Sex	*n* (%)
Male	21 (55.3)
Female	17 (44.7)
Median age (SD)	70.3 (19.4)
Classification	*n* (%)
HA-CDI	23 (60.5)
CA-CDI	12 (31.6)
Indeterminate	3 (7.9)
Median length of stay in days (SD)	17.5 (22.8)
Principle diagnosis on primary admittance^1^	*n* (%)
Enterocolitis	3 (7.9)
Malignancy	3 (7.9)
Skin and soft tissue infection	2 (5.3)
Bone fracture	2 (5.3)
Pancreatitis	1 (2.6)
Urinary tract infection	1 (2.6)
Crohn’s disease	1 (2.6)
Hypogammaglobulinemia	1 (2.6)
Cerebral infarction	1 (2.6)
Intracranial abscess	1 (2.6)
Poisoning	1 (2.6)
Kidney failure	1 (2.6)
Cellulitis	1 (2.6)
Varicose veins	1 (2.6)
Epilepsy	1 (2.6)
Zoster	1 (2.6)
Thyrotoxicosis	1 (2.6)
Diabetes	1 (2.6)
Neutropenia	1 (2.6)
HIV	1 (2.6)
Myeloid leukaemia	1 (2.6)
Osteoarthritis	1 (2.6)
Unspecified	10 (26.3)

^1^International Classification of Diseases (ICD-10)

### Molecular epidemiology of *C. difficile* strain population

The most prevalent RTs identified in the 94 isolates (Fig. 1) were RT014/020 (32.9%), RT002 (19.1%) and RT056 (11.7%), shared by 14 (36.8%), seven (18.4%) and four (10.5%) patients, respectively. Other notable RTs included 251 (5.3%), 078 (3.2%) and 017 (2.1%). There were no RT027 strains. All strains harboured one or more toxin genes (*tcdA*, *tcdB*, *cdtA/B*), with the genotype A^+^B^+^CDT^−^ most prevalent (89.4%), followed by eight A^+^B^+^CDT^+^ strains (8.5%) and two A^-^B^+^CDT^−^ strains (2.1%). Toxin genotypes determined by PCR and WGS were 100% concordant.

**Fig. 2:**
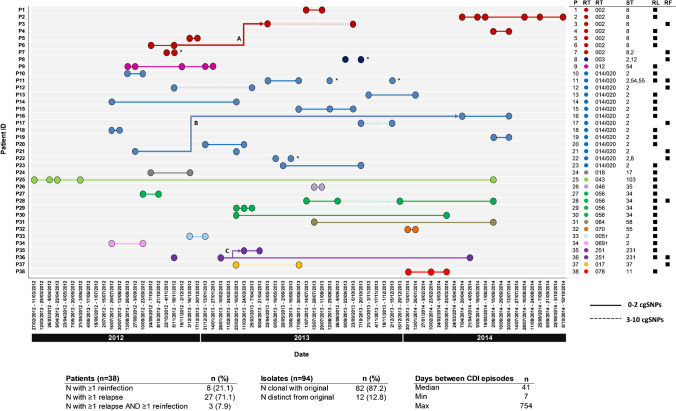
Temporal and genetic relatedness of *C. difficile* isolates from patients with rCDI in WA investigated by WGS. Data is shown for 94 isolates collected from 38 patients (P1–38) over 32 months (Feb 2012–Oct 2014). Clinical CDI episodes are represented as coloured circles according to RT as indicated in the key. Relatedness between isolates and classification of recurrent disease as relapse (RL) or reinfection (RF) is evaluated by differences in cgSNPs using thresholds proposed by Eyre et al. [17]. Isolates separated by ≤ 2 cgSNPs represent clonal transmission and are thus characterised as a relapse (indicated by a bold line between isolates). Isolates separated by 3–10 cgSNPs are not clonally related and are thus characterised as reinfection (indicated by the dashed line between isolates). For some patients, there was significant genetic variation between isolates of the same RT (indicated by unconnected by either a bold or dashed line) meaning > 11 cgSNPs between isolates, or in some cases (P7, P8, P11, P22), one or more isolates belong to a different ST than the original isolate (*). In both scenarios, the rCDI is characterised as reinfection. Bold arrows connecting isolates from different patients indicate clonal transmission of *C. difficile* between epidemiologically unrelated cases (A–C, patients P6/P3 (RT002); P21/16 (RT014/020); and P36/P35 (RT251). Novel RTs QX005 and QX069 are indicated with (†)

A total of 13 STs were identified comprising *C. difficile* lineages spread across four evolutionary clades (C1, C2, C4 and C5, Table 2). The most prevalent STs were ST2 (36.2%), ST8 (19.1%) and ST34 (11.7%). Overall, the MLST predicted from WGS largely supported the initial RT data in confirming CDI relapse, i.e. the *C. difficile* strains causing the initial and recurrent infections were the same ST (Fig. 2). Moreover, with four exceptions, STs were congruent with previously reported MLST–RT correlations. Of the 38 patients with rCDI, four (10.5%) yielded strains of *C. difficile* from their initial and recurrent episodes that despite sharing the same RT, had a different ST. For three of these four patients (P7; RT002, initial ST8, recurrence ST2), (P8; RT003, initial ST2, recurrence ST12) and (P22; RT014/020, initial ST2, recurrence ST8), rCDI episodes can be definitively classified as reinfection rather than relapse. For P11, who experienced four episodes of CDI over 111 days, a single RT was identified (RT014/020), yet three different STs were found across the four episodes, indicating both a relapse (initial ST2, first recurrence ST2) and reinfection (second recurrence ST54, third recurrence ST55).

**Table 2:** *C. difficile* multi-locus sequence types identified in the study

Sequence type	N isolates	% isolates	Evolutionary clade
2	34	36.2%	1
8	18	19.1%	1
34	11	11.7%	1
54	6	6.4%	1
231	5	5.3%	2
103	5	5.3%	1
11	3	3.2%	5
55	3	3.2%	1
17	2	2.1%	1
35	2	2.1%	1
37	2	2.1%	4
58	2	2.1%	1
12	1	1.1%	1

### Core genome-based differentiation of CDI relapse from reinfection

Within each ST group, pairwise cgSNP analysis was performed and established thresholds based on the predicted within-host evolutionary rate for *C. difficile* [17] were applied to distinguish CDI relapse from reinfection. A summary of the temporal and genetic relatedness of all 94 isolates is presented in Fig. 2. Of the 38 patients, 27 (71%) experienced relapses i.e. recurrences were caused by clonal strains with ≤ 2 cgSNPs difference. Eight patients (21.1%) experienced reinfections, i.e. recurrences were caused by non-clonally related strains with ≥ 3 cgSNPs difference and the remaining three patients (P11, P28 and P36) experienced both CDI relapse and reinfection.

**Fig. 3:**
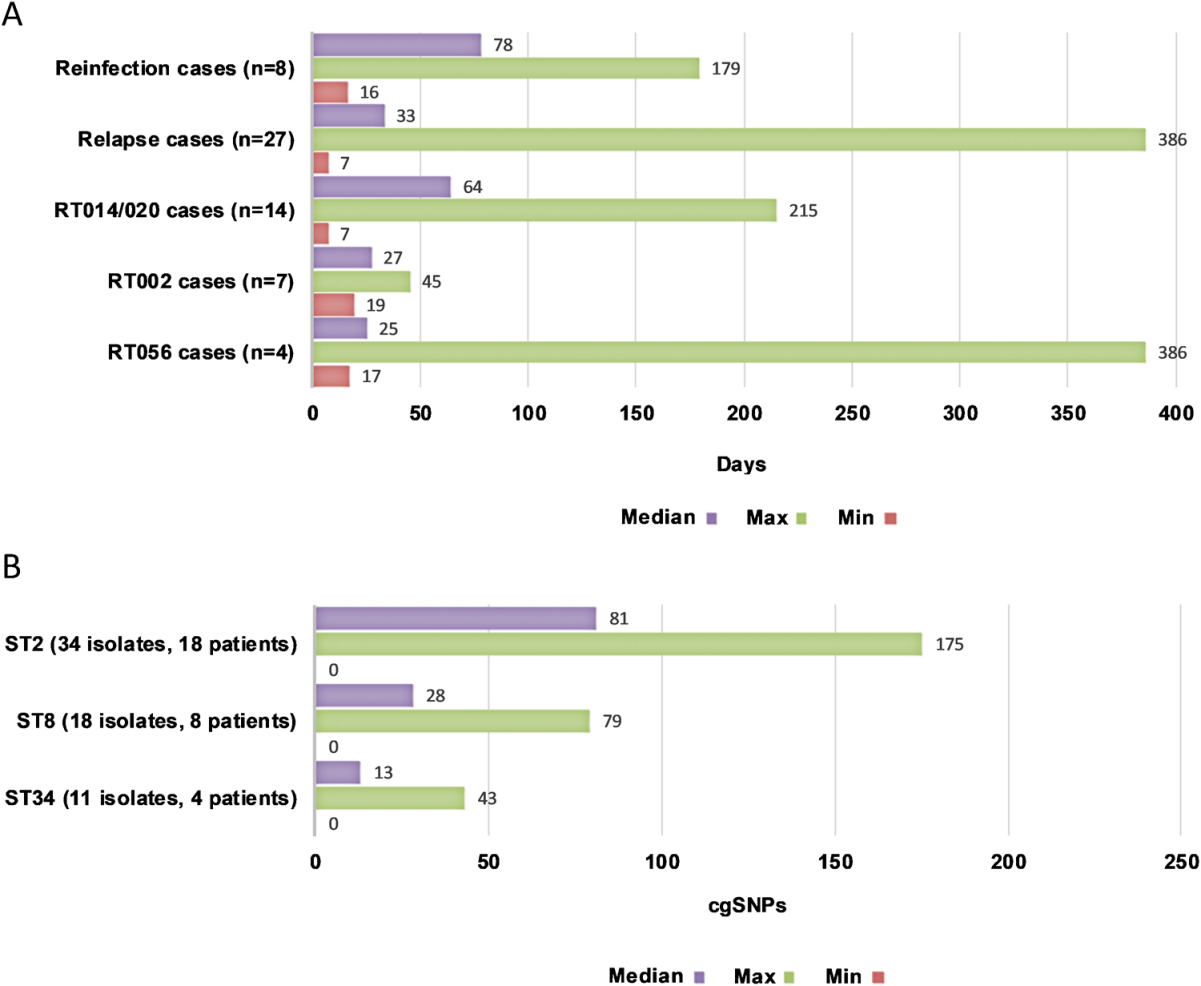
Temporal analysis of rCDI episodes and strain heterogeneity. **A** Days between CDI episodes for relapse cases, reinfection cases and cases associated with the most prevalent RTs 014/020, 002 and 056. **B** Comparative strain heterogeneity for STs 2, 8 and 34

**Fig. 4:**
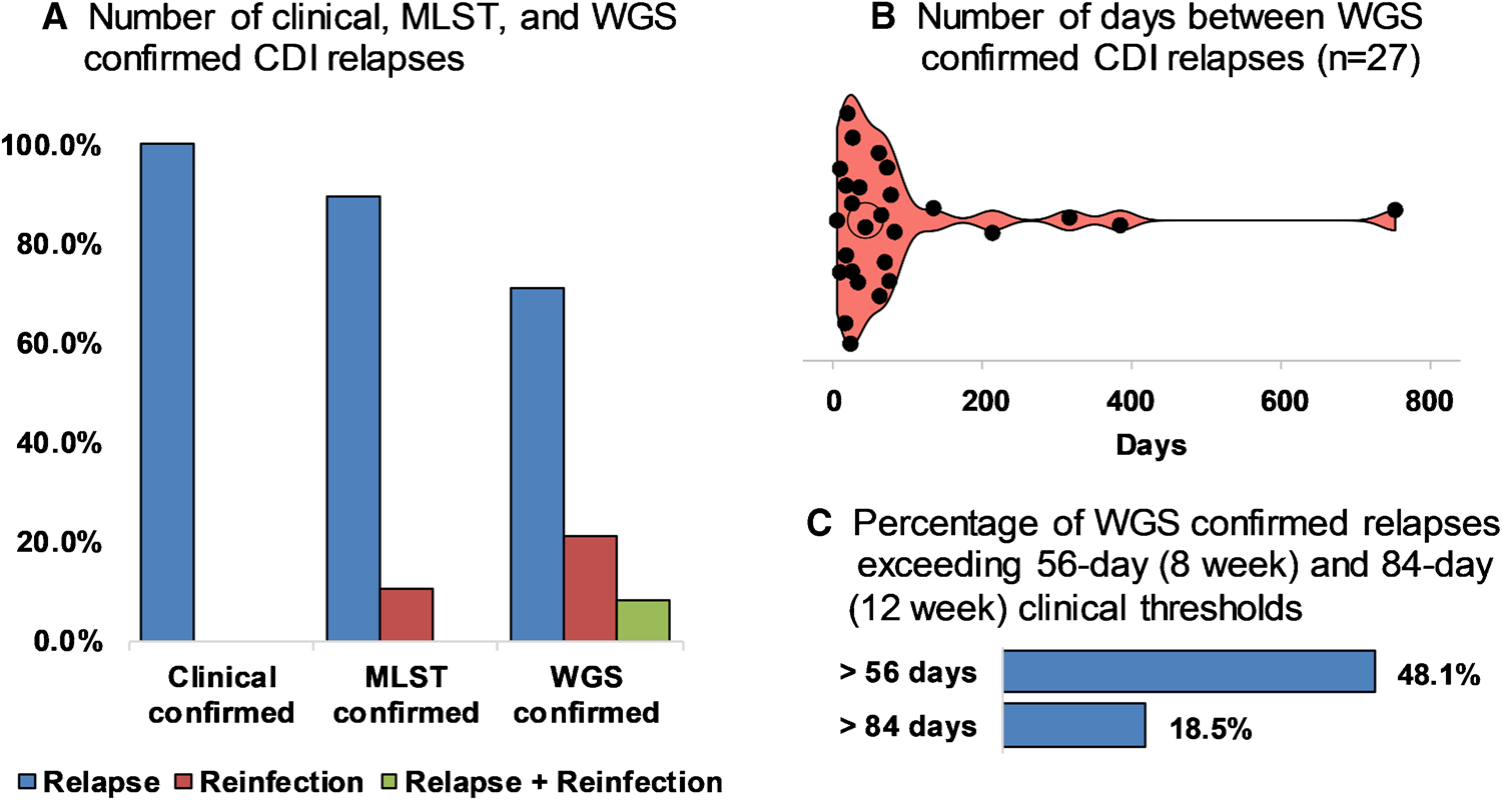
WGS confirmed CDI relapses. **A** Comparison of clinical, MLST and WGS confirmed CDI relapses. **B** Violin plot illustrating the temporal distribution of WGS confirmed relapses. **C** WGS confirmed relapses occurring after 8-week (56-day) and 12-week (84-day) thresholds

When analysed by RT and ST, there were no significant differences in the proportion of defined CDI relapses or reinfections. Moreover, genetic diversity within STs was low, even across epidemiologically distinct patients (Fig. 3). The most heterogeneous lineages were ST2 with 34 strains from 18 patients differing by a median of 81 cgSNPs (range 0–175), followed by ST8 with 18 strains from eight patients differing by a median of 28 cgSNPs (range 0–79). The time between CDI episodes for relapse cases, reinfection cases and the cases associated with the most prevalent RTs is shown in Fig. 4. Of the 27 patients with WGS-confirmed CDI relapse, 13 (48.1%) suffered one or more episodes that occurred outside the accepted 8-week (56-day) timeframe of the definition of recurrent CDI [9]. Most notable of these were P14 (215 days between WGS-linked recurrences), P30 (386 days), P31 (318 days) and P25 (754 days) (Fig. 4).

### A clonal outbreak of clade 2 virulent RT251 in 2 Australian States

**Fig. 5:**
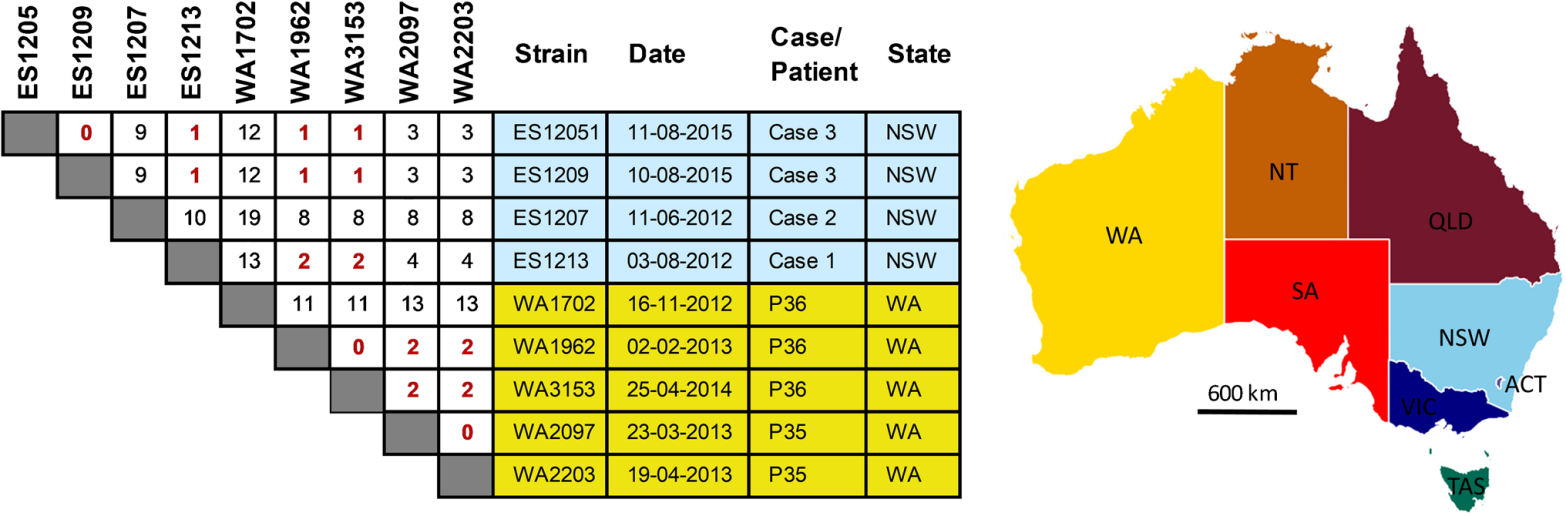
cgSNP analysis of RT251 cluster. Pairwise cgSNP analysis for nine *C. difficile* RT251 strains isolated from patients with rCDI in WA (two patients, five isolates from this study) and from previously reported cases in New South Wales (three cases, four isolates) [22]. Red numbers indicate plausible clonal transmission (≤ 2 cgSNPs between strains). Australian States: NSW, New South Wales; SA, South Australia; VIC, Victoria; QLD, Queensland; TAS, Tasmania. Australian Territories: NT, Northern Territory; ACT, Australian Capital Territory

Core genome analysis identified three potential transmission events among epidemiologically unrelated patients (Fig. 2). Genetically indistinguishable clones of RT002 and RT014/020 were found in patients P6/P3 and P21/P16, respectively. Interestingly, patients P35 and P36 harboured a total of five *C. difficile* strains belonging to virulent clade 2 lineage RT251, a close relative of RT027. Of these strains, four were clonally related (P35 initial and recurrence, and P36 first and second recurrence) with the fifth, the initial case for P36, differing by 11-13 cgSNPs (Fig. 2). Comparative analysis with four further RT251 strains derived from three patients in a previously published outbreak in New South Wales in 2012–2015 [22] was performed (Fig. 5). All nine RT251 strains were highly related (0–19 cgSNPs, median 3 cgSNPs) and determined to be likely part of the same outbreak.

### Characterising the AMR repertoire of *C. difficile* strains causing rCDI

Using in vitro antimicrobial susceptibility testing and WGS, we investigated the AMR phenotype and genotype of strains isolated from these 38 patients with rCDI. Summary antimicrobial susceptibility data (MIC range, MIC_50_, MIC_90_ and MIC breakpoints) for the 94 *C. difficile* strains are presented in Table 3. All strains were fully susceptible to first-line therapies vancomycin (MIC_90_ 0.5 mg/L) and metronidazole (MIC_90_ 0.25 mg/L) with varying levels of non-susceptibility (resistant and intermediate breakpoints) observed for moxifloxacin (12%), tetracycline (1%) and clindamycin (47%). No multidrug resistance was observed.

**Table 3:** Antimicrobial susceptibility for 94 *C. difficile* strains

Antimicrobial	MIC range (mg/L)	MIC_50_/MIC_90_ (mg/L)	Breakpoint (mg/L)			Susceptibility (%)		
			S	I	R	S	I	R
Vancomycin	0.19–0.75	0.38/0.5	≤ 2	-	≥ 2	100	-	0
Metronidazole	< 0.016–1.5	0.094/0.25	≤ 2	-	≥ 2	100	-	0
Moxifloxacin	0.25–> 32	1/> 32	≤ 2	4	≥ 8	88	0	12
Tetracycline	< 0.016–6	0.023/0.47	≤ 4	8	≥ 16	99	1	0
Clindamycin	0.125–> 256	2/> 256	≤ 2	4	≥ 8	53	26	21

MIC breakpoints for vancomycin and metronidazole were based on EUCAST recommendation [20]. MIC breakpoints for moxifloxacin, tetracycline and clindamycin were based on CLSI recommendations [21]

**Fig. 6:**
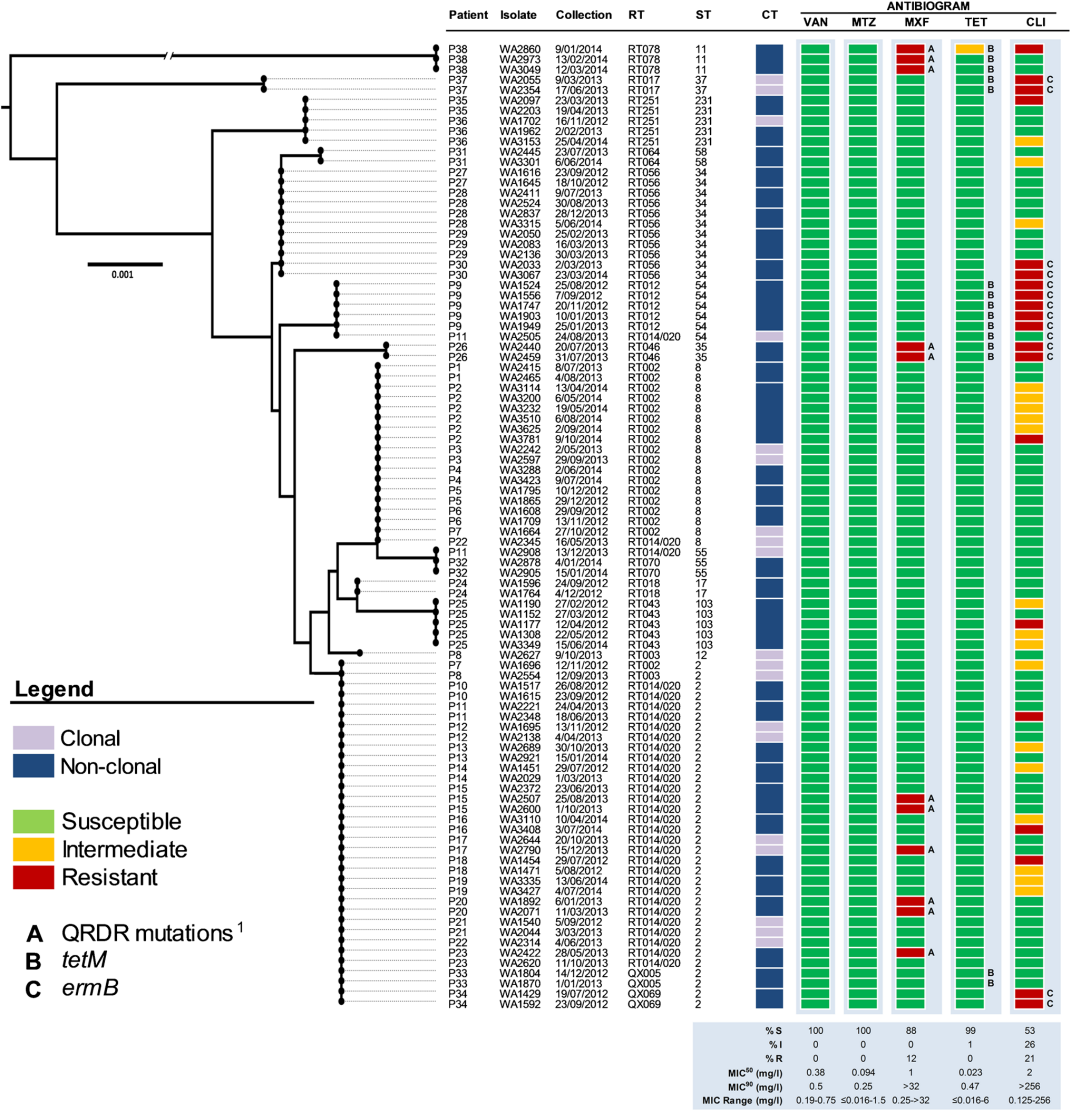
Comparative MLST and AMR analysis. Neighbour-joining MLST phylogeny for 94 study isolates (15 RTs, 13 STs) built from concatenated housekeeping gene allele sequences (7 loci, 3501 bp). The scale bar shows the number of SNPs per site. For each isolate/patient, clonal transmission (CT, ≤ 2 cgSNPs between strains) is indicated as per the legend. For each isolate, the antimicrobial phenotype is indicated as per the legend and is based on CLSI and EUCAST criteria (see methods). Antimicrobials are abbreviated as follows: VAN, vancomycin; MTZ, metronidazole; MXF, moxifloxacin; TET, tetracycline; CLI, clindamycin. The presence or absence of antimicrobial resistance loci is indicated as per the legend. ^1^QRDR mutations comprise one or more of Thr82Ile (GyrA) and Ser366Val, Ser416Ala, Asp426Asn, Asp426Val, Arg447Lys, Glu466Val (GyrB). Other AMR loci identified (but not shown) include *cfr* (linezolid resistance, P35 isolates WA2440 and WA2459); *aac6-aph2* (aminoglycoside resistance, P37 isolates WA2055 and WA2354); and *ant(6)-Ia* (aminoglycoside resistance, P38 isolates WA2860, WA2973 and WA3049)

Eleven isolates (12%) were moxifloxacin resistant (MIC ≥ 8 mg/L, RTs 078, 046 and 014/020, Fig. 6), all with concordant genotypes defined by the presence of at least one nonsynonymous mutation in the quinolone resistance determining region (QRDR) of GyrA (Thr82Ile) and GyrB (Ser366Val, Ser416Ala, Asp426Asn, Asp426Val, Arg447Lys and Glu466Val). Twenty isolates were clindamycin resistant (MIC ≥ 8 mg/L, RTs 002, 012, 014/020, 017, 043, 056, 078 and 251, Fig. 6); however, only 13 of these (65.0%) possessed concordant genotypes defined by the presence of either *ermB* (encoding a 23S rRNA methyltransferase) or other known macrolide or lincosamide resistance loci (e.g. *ermTR*, *ermC*). A single RT014/020 isolate harboured *ermB* but remained susceptible in vitro. Concordance between tetracycline phenotype and genotype was poor, with *tetM* genes found in 14 susceptible isolates (MIC 0.016–4 mg/L, RTs 012, 017, 046 and 078) and one intermediate isolate (MIC 6 mg/L, RT014, Fig. 6). Other AMR loci identified include *cfr* (linezolid resistance, P35, *n* = 2 isolates); *aac6-aph2* (aminoglycoside resistance, P37, *n* = 2 isolates); and *ant(6)-Ia* (aminoglycoside resistance, P38, *n* = 3 isolates).

### Changes in clonal AMR phenotypes and genotypes between recurrent episodes

For several WGS-inferred relapses, significant changes in AMR phenotype and genotype were observed relative to the initial episode (Fig. 6). Different moxifloxacin (MXF) MICs and QRDR genotypes were found for clonal RT014/020 isolates from P15 (acquisition of Asp426Asn mutation in GyrB, MXF MIC change 0.75 mg/L to ≥ 32 mg/L), P17 (acquisition of Thr82Ile mutation in GyrA, MXF MIC change 0.75 mg/L to ≥ 32 mg/L) and P23 (loss of Ile82Thr mutation in GyrA, MXF MIC change ≥ 32 mg/L to 0.75 mg/L). Despite the absence of *ermB*, different clindamycin (CLI) MICs were found for clonal isolates from P35 (RT251, CLI MIC change > 256 mg/L to 1.5 mg/L) and P38 (RT078, CLI MIC change 8 mg/L to 3 and 2 mg/L in the second and third episodes, respectively).

### Investigation of potential zoonotic or environmental origins

Comparative analysis of strains of prominent RTs with recent animal and environmentally derived isolates was performed. A total of 47 isolates from STs 54 (RT012, *n* = 5), 2 (RT014/020, *n* = 31), and 34 (RT056, *n* = 11) were compared by cgSNP typing to genomes of matching *C. difficile* STs isolated from recent studies in WA [23–25] (Table 4). The number of cgSNPs identified for the ST54, ST2 and ST34 groupings ranged from 43–989, 5–416 and 8–43, respectively. Whilst there was no evidence of clonal transmission between isolates derived from the environmental and human origin (defined as ≤ 2 cgSNPs), there were two instances of genomic clustering of strains (defined as ≥ 3 and ≤ 10 cgSNPs) from these sources (Table 4). In the first instance, the clonal RT014/020 isolates from P19 (WA3335 and WA3427) differed by just 5 cgSNPs from 5 clonal RT014/020 isolates obtained from lawn in urban WA in 2016. In the second instance, the clonal RT056 isolates from P27 (WA1616 and WA1645), P29 (WA2050, WA2083 and WA2136) and P30 (WA2033 and WA3067), differed by just 8-9 cgSNPs from three clonal RT056 isolates obtained from organic potatoes in urban WA in 2016.

**Table 4:** Evolutionary relatedness of prominent ribotypes recovered from patients with rCDI and the environment in WA

Environmental source	cgSNP differences between patient isolates (n) and environmental sources of *C. difficile*																		
	P9 (5)	P10 (2)	P11 (4)	P12 (2)	P13 (2)	P14 (2)	P15 (3)	P16 (2)	P17 (2)	P18 (2)	P19 (2)	P20 (2)	P21 (2)	P22 (2)	P23 (2)	P27 (2)	P28 (4)	P29 (3)	P30 (2)
RT012-ST54																			
Lawn (*n* = 12016, WA)	985																		
Beetroot (*n* = 12014, WA)	989																		
Compost (*n* = 12015, WA)	43																		
RT014/020-ST2																			
Lawn (*n* = 52016, WA)		38	19	416	31	19	40	11	408	23	**5**	12	13	35	36				
RT056-ST34																			
Sheep manure (*n* = 12015, WA)																10	41	18	11
Soil conditioner (*n* = 12015, WA)																8	41	16	11
Lawn (*n* = 12016, WA)																13	43	18	13
Organic carrots (*n* = 12015, WA)																14	43	18	13
Organic potatoes (*n* = 32016, WA)																**8**	41	**9**	**9**

*WA* Western Australia, Numbers in red indicate a high degree of genetic similarity

## Discussion

The purpose of this study was to use WGS to better define the epidemiology and transmission dynamics of rCDI in WA. We found the most common *C. difficile* strains causing rCDI in this cohort were toxigenic STs 2 (RT014), 8 (RT002) and 34 (RT056), together accounting for over 65% of cases. These data are consistent with genomic-based studies from the USA [26, 27] and Europe [14, 17, 28] where these RTs are among the most common causes of rCDI. Moreover, they are consistent with our earlier molecular-based analysis of 551 patients with rCDI in WA [29]. *C. difficile* RT014 spans multiple STs in clade 1 and is one of the most successful *C. difficile* lineages worldwide [16, 30, 31]. It has been a leading cause of CDI in Australia for many years [16], accounting for ~ 25% of CDI cases nationally each year, and is well established in Australian pig herds [32]. *C. difficile* RTs 002 (ST8) and 056 (ST34) are also common RTs circulating in Australia [16] and across Europe [30]. The epidemic RTs 078 and 027 have been linked to high rates of rCDI [26, 28]; however, we found a low prevalence of RT078 (8%) and no RT027 which was not unexpected as these lineages are not commonly found in Australia [18]. While *C. difficile* RT027 was absent, the closely related RT251 was found in two cases with interesting epidemiology (discussed below).

Conventional typing methods for *C. difficile* such as PCR ribotyping, pulsed-field gel electrophoresis and MLST cannot adequately distinguish relapse from reinfection [27]. These approaches frequently lead to an overestimation of relapses and an underestimation of reinfections [13, 17]. With its ability to detect fine-scale within-strain diversity, WGS can better distinguish relapse from reinfection and identify putative patient-to-patient strain transmission events [14]. Using WGS, we found the majority (71%) of CDI recurrences were due to relapse with the same antecedent *C. difficile* strain, whereas reinfection with a new genetically distinct strain accounted for a smaller fraction (21%) of recurrences, with three patients (8%) experiencing both CDI relapse and reinfection. Using WGS, Sim et al. [26] determined that 15% of rCDI cases in a single US hospital were due to reinfection by a new strain, and one-third of such cases would have been misclassified as relapse based solely on ribotyping. Here, we found the number of WGS-confirmed relapses was ~ 28% and 18% fewer than identified by clinical (RT)-based and MLST-based approaches, respectively. Our findings indicate relapsing infections with the same endogenous initial *C. difficile* strain rather than reinfection with a new exogenous strain is the main driver of rCDI in our setting. This is consistent with the paradigm that *C. difficile* spores persist in the gut during antimicrobial therapy and vegetate after cessation of treatment. Relapse signifies incomplete eradication of the organism—suppression of initial infection (partial cure) or a persistent reservoir in the gastrointestinal tract, or in the environment (discussed below). Conversely, rather than a failure of initial treatment to eradicate the causative strain, reinfection signifies an individual with a higher propensity of developing CDI (slow reconstitution of host microbiota) and failure to reverse the effects of predisposing risk factors (e.g. re-exposure to *C. difficile* spores in the community).

An accurate distinction between relapse and reinfection is important. It allows for the evaluation of risk factors and effective guidance of patient management and treatment policies [2–4, 12]. In the literature, the proportion of relapses ranges from 52 to 88% compared with 12% to 42% for reinfections [8, 13, 26–29]. Such variation can be a result of factors including local strain epidemiology, differences in patient populations, infection prevention practices, typing methods (as detailed above) and, most notably, rCDI case definitions. Current clinical guidelines [9] define relapse as a CDI episode occurring between 2 and 8 weeks after the successful resolution of symptoms of a previously confirmed CDI episode. However, studies have shown that the 8-week interval does not allow sufficient discrimination of relapse and reinfection; for the majority of relapsing CDI cases confirmed by conventional PCR ribotyping, the time between initial and second episode ranged from 4 to 26 weeks (average 12 weeks) [12, 29]. Acknowledging this limitation, studies from the USA [33], Europe [11] and Australia [29] have used a longer 12-week cut-off. In this study, 48% of WGS-confirmed relapses occurred beyond the 8-week (56-day) cut-off and, of these, 19% occurred beyond 12 weeks (84 days). Together, these data indicate that both the 8- and 12-week intervals fail to adequately distinguish reinfection from relapse and suggest that ‘time after the previous episode’ may not be a good indicator of relapse or reinfection. We also found four CDI relapses that occurred beyond 20 weeks, an optimal cut-off recommended by studies in Switzerland [8] and the USA [34]. One of these relapses involved a *C. difficile* RT043 (ST103) clone persisting in a patient for over 2 years (754 days) which exceeds current definitions for recurrence by > 100 weeks. Although rare, there have been reports of apparent CDI relapse caused by indistinguishable *C. difficile* strains collected over 191 [28] and 561 [35] days. It is plausible that rather than persistence in the host, lengthy intervals between initial infection and relapse could be due to a new infection with a genetically identical strain in the community or environment. To determine this, bacterial culture must be performed after each CDI episode to confirm whether the patient is cured microbiologically.

Initial infection with epidemic *C. difficile* RT027 (NAP1/BI) strains is associated with more frequent recurrences due to a higher rate of therapeutic failure [7]. At the height of the RT027 outbreak in Canada in the early 2000s, which was associated with greater complications of CDI (shock, need for colectomy, megacolon, perforation) and significant patient mortality, ~ 9% of patients with at least one CDI recurrence died within 30 days of recurrence [36]. We found two epidemiologically unrelated patients with near-identical (2 cgSNPs) strains of *C. difficile* RT251, a close relative of RT027, with a similar virulence phenotype [22]. *C. difficile* RT251 clones from patients in WA differed by only 1–19 cgSNPs from three clinical cases in New South Wales in 2012, two with multiple recurrences, and the third, fatal [22]. A defining characteristic of the earlier cases was that they occurred in young previously healthy individuals who were infected in a community setting. Here, P36 (aged 56) had community-onset CDI, whereas P35 (aged 97) had acquired CDI in the hospital. Our temporal genomic analyses suggest potential transmission of the RT251 strain from P36 to P35 in early 2012. Moreover, the clustering of RT251 clones indicates these WA cases were part of the broader, likely community-driven, *C. difficile* RT251 outbreak occurring in the Eastern States of Australia at that time [22].

Australia has seen CDI become a significant problem in the community setting with hallmarks of a ‘One Health’ aetiology [37]. In the USA, patients with community-onset of rCDI, and harbouring genetically similar strains of *C. difficile* (by cgMLST), shared the same postcode, suggesting a potential common community reservoir [27]. Moreover, CA-CDI recurrences are ~ 20% lower than in HA-CDI [31], the difference attributed to younger patient age and reduced exposure to healthcare facilities in the CA-CDI population. Our study found a close genetic relationship between *C. difficile* isolates causing rCDI and *C. difficile* isolates derived from food and environmental origin. These clusters included *C. difficile* RTs 014/020 and 056 from humans with rCDI in Perth and samples of new roll-out lawns and organic potatoes, respectively, both from metropolitan areas of Perth. Both RTs are toxigenic and among the leading strains causing CDI in Australia [16] and well-established in Australian livestock [37]. Despite no evidence of clonal *C. difficile* transmission, our findings further support the paradigm that animals and the environment are playing a critical, yet still underappreciated, role in *C. difficile* transmission to humans [37]. Longitudinal genomic- and One Health-focused surveillance of *C. difficile* is needed to fully understand the epidemiology and burden of rCDI in healthcare and community settings.

AMR is a key driver of *C. difficile* epidemiology with CDI outbreaks linked to the evolution of resistance to clindamycin (RT017), fluoroquinolones (RT027) and tetracycline (RT078) [38]. The *C. difficile* population in this study did not show extensive levels of AMR and no MDR was found. Resistance to moxifloxacin was found in 12% of isolates and attributed to known nonsynonymous mutations in the QRDR of GyrA and GyrB. Historically, Australia has had a low prevalence of fluoroquinolone resistance due to (i) the absence of epidemic RT027, and (ii) the conservative use of fluoroquinolones in Australia. Although lower than seen in Asia-Pacific countries (44.4%) and the USA (37.5%), the prevalence of moxifloxacin resistance in this study (12%) was considerably higher than reported in our national *C. difficile* AMR surveillance program [39] where 3.5% (38/1091) of *C. difficile* isolates collected between 2015 and 2018 in five Australian states were resistant to moxifloxacin (*χ*^2^, *p* = 0.0001). Moreover, within-host strain evolution was observed for some WGS-confirmed relapse cases, characterised by the acquisition of moxifloxacin resistance in two patients (both ~ 8 weeks between episodes with at least a 42.7-fold increase in MIC) and the loss of resistance in another patient (~ 18 weeks between episodes with at least 42.7-fold decrease in MIC).

As we have previously reported [18], there was poor concordance between phenotype and genotype for both clindamycin and tetracycline. Over a third of MLS_B_+ isolates did not harbour any known macrolide or lincosamide resistance determinant, suggesting a yet-to-be-identified resistance mechanism. Conversely, *tetM* genes were present in 15% of tetracycline-susceptible *C. difficile* isolates, indicating genetic or epigenetic barriers to the expression of tetM protein. All *C. difficile* strains were fully susceptible to vancomycin and metronidazole, consistent with recent national surveillance of > 1000 *C. difficile* isolates in Australia [39]. Despite the initial resolution of symptoms, significant recurrence rates are seen post-treatment with vancomycin and metronidazole. Consequently, the latest CDI treatment guidelines recommend fidaxomicin as the preferred agent for the initial episode of CDI and the first recurrence [9]. Despite clear long-term therapeutic benefits, fidaxomicin has not been widely adopted into clinical practice in Australia, principally due to substantially high pharmacy costs compared to vancomycin (USD $92/dose vs. USD $5/dose, respectively) [40]. Indeed, during the study period (2012–2014), fidaxomicin use in Australia was negligible and, at the time, CDI treatment almost exclusively relied on vancomycin and metronidazole. While similar to vancomycin in effectiveness for treatment of the rCDI, fidaxomicin demonstrates superior efficacy in achieving a sustained cure and significantly reduces CDI recurrences as it spares critical components of the host gut microbiota [9, 13]. In hindsight, it is tempting to suggest that some of the 38 recurrence cases identified in this study could have been prevented by treating the initial CDI episode with fidaxomicin.

Our study has important limitations. First, because WGS is performed on a single colony, we may have overlooked the presence of multiple strains during the initial infection. Significant within-host genetic diversity could impede the identification of inter-patient transmission events. Second, in our study setting, only diarrhoeal specimens were tested for CDI; thus, we were unable to determine from the data whether CDI was resolved after each episode. We also lacked clinical data to confirm the presence (or absence) of antimicrobial selection pressure which could account for the observed gain/loss of moxifloxacin resistance in some isolates. Finally, we did not include or rule out any other possible sources for *C. difficile* hospital transmission such as asymptomatic carriers or the hospital environment [41].

In summary, this is the first study in Australia to use WGS to distinguish CDI relapse from reinfection. Our findings suggest that current 8- and 12-week clinical intervals fail to distinguish between relapse and reinfection and require revision. WGS-based infection tracking of the persistence and spread of *C. difficile* within healthcare facilities could enhance infection prevention and control, and patient management for CDI. For those patients with WGS-confirmed relapse, new therapeutic options may be considered, such as fidaxomicin or faecal microbiota transplantation [9]. Conversely, patients with WGS-confirmed reinfection could indicate the current infection prevention and control strategies in place have failed, and alternative disinfection measures should be considered. Our study also shows a potential, underappreciated role of the environment as a source of *C. difficile* in patients with rCDI. There is an urgent need for locally and nationally coordinated One Health-focused genomic surveillance of *C. difficile*, to better understand CDI epidemiology, enhance CDI control strategies and improve patient outcomes.

## Data Availability

Whole-genome sequence data generated in this study have been submitted to the NCBI Short Read Archive under study PRJNA880992 (accessions SRR21600731 - SRR21600623). Supplementary data including a detailed summary of strains and associated epidemiological data, along with assembled genome sequences is hosted at 10.6084/m9.figshare.20579976.v3.

## Code availability

All code used in this study is publicly available. Version numbers, websites and references are provided in Materials and Methods.
